# Strengthening tobacco control policy: using plain packaging to reduce product appeal and enhance public awareness

**DOI:** 10.3389/fpubh.2026.1781256

**Published:** 2026-03-09

**Authors:** Ho Cheung William Li, Wei Xia, Hong Chen, Xinyi Xu, Laurie Long Kwan Ho, Hoi Yan Cora Mok, Oi Kwan Joyce Chung

**Affiliations:** 1 Nethersole School of Nursing, The Chinese University of Hong Kong, Hong Kong, Hong Kong SAR, China; 2 School of Nursing, Sun Yat-sen University, Guangzhou, China; 3 School of Nursing, The Hong Kong Polytechnic University, Hong Kong, Hong Kong SAR, China

**Keywords:** health policy, perceptions, pictorial health warnings, plain packaging, tobacco control

## Abstract

**Background:**

Tobacco use remains a leading cause of preventable morbidity and mortality worldwide. While smoking prevalence has recently declined in Hong Kong, progress has slowed. Plain packaging may reduce product appeal and enhance the impact of pictorial health warnings (PHWs), supporting reduced tobacco use.

**Methods:**

A cross-sectional study was conducted from August to November 2023 among 1,256 adult smokers in Hong Kong. Participants were recruited at outdoor public smoking hotspots and shown branded and plain cigarette packs with 85% PHWs. Perceptions of pack attractiveness, noticeability of PHWs, smoking-related harm, behavioural intentions, and support for plain packaging were assessed through structured interviews. Quantitative data were weighted by age and sex, and qualitative responses were analysed thematically.

**Results:**

Most participants perceived plain packs as less attractive (79.8%) and PHWs on plain packs were more noticeable (57.5%) than branded packs. About 45.1% perceived plain packs as disgusting, and 28.0% agreed they better conveyed severe smoking harms than branded packs. While only 17.3% reported plain packs may reduce their intention to smoke, 33.1% reported that it could deter youth from smoking. Support for plain packaging was moderate, with 35.7% in favour and 34.5% neutral. The qualitative findings reinforced these perceptions.

**Conclusion:**

Smokers perceived plain packaging as less attractive, the PHWs on it as more noticeable, and potentially useful in preventing smoking initiation in youth. These findings support the integration of plain packaging into Hong Kong’s tobacco control strategy and offer valuable insights for jurisdictions considering similar legislation.

## 1 Introduction

Tobacco use remains one of the leading causes of preventable morbidity and mortality globally, underscoring the need for robust, evidence-based policy interventions (1). In Hong Kong, the prevalence of smoking has remarkably remained low among regions in the world (2), which can largely be attributed to the sustained efforts by the Hong Kong government and tobacco control advocates over the past four decades (3). These efforts include substantial increases in tobacco taxation, the enactment and enforcement of comprehensive legislation, and the implementation of widespread anti-smoking campaigns.

According to the 2024 Thematic Household Survey, the prevalence of daily cigarette smoking among individuals aged 15 years and older in Hong Kong declined steadily from 23.3% in 1982 to 9.1% in 2023 (3). Despite this notable progress, the pace of decline in smoking prevalence – both globally and locally – has slowed in recent years, which signals the need for more innovative and stringent policy measures to reduce tobacco use further and protect public health.

In alignment with the WHO’s 2025 World No Tobacco Day theme, ‘Unmasking the Appeal: Exposing Industry Tactics and Tobacco and Nicotine Products’, the introduction of plain packaging represents a promising next step in countering the tobacco industry’s strategic use of branded packaging to mislead the public by downplaying the harms of tobacco products. By standardising the appearance of tobacco products and eliminating branding elements, plain packaging initiatives aim to diminish the visual appeal of smoking, particularly among youth, while simultaneously reinforcing public awareness of the serious health risks associated with smoking.

Pictorial health warnings (PHWs) that illustrate the harmful effects of tobacco use can elicit strong negative emotions, including fear, guilt, and disgust (4). Compared to text-only warnings, PHWs are more effective in deterring smoking initiation among non-smokers and stimulating intentions to quit among smokers (5, 6). In 2008, the WHO Framework Convention on Tobacco Control recommended that PHWs should cover ≥50% of the front and back of each cigarette packet (7). As of October 2021, at least 122 countries/jurisdictions have complied with this regulation, with 10 jurisdictions extending the legislature by requiring at least 85% of the cigarette packets to be covered with PHWs, including Hong Kong (8).

Hong Kong has required 50% covered with PHWs since 2007, which was increased to 85% in 2017 (9). Despite the smoking prevalence in Hong Kong has steadily decreased (10), there is still quite a gap in achieving the Hong Kong government’s target of lowering smoking prevalence to 7.8% by 2025 (11). In addition, a local survey in 2018 found that current smokers’ awareness of point-of-sale tobacco displays increased after the implementation of larger PHWs (9). This increased awareness was likely due to the tobacco industry’s counter tactics: for example, some shops would cover up the PHWs that only the logos, colours, and design of the cigarette brands were displayed (9). Cigarette packaging is the primary marketing vehicle for tobacco industry to promote brand imagery and establish brand loyalty; in addition, and tactful packet designs can mislead smokers into thinking that cigarettes may be ‘safer’ (12, 13).

In 2012, Australia was the first country worldwide to mandate plain tobacco packaging. After 3 years following the implementation, plain packaging was attributable to a 0.55% decline in smoking prevalence in Australia, approximately a quarter of the total decline (2.2%) (14). Compared with branded packaging, plain packaging is perceived as having less attractiveness, inferior taste, lower quality, increased tar content and increased health risks (15–19). Moreover, plain packaging may draw more attention to the PHWs and consequently increase negative pack perceptions when adopted in combination (15, 20). However, research findings on the perceived influence of plain packaging on smoking behaviour have been mixed. While some studies found that exposure to plain packaging increased current smokers’ intention to quit and reduced purchase intention, other studies found no significant perceived effect on their smoking behaviours (17, 19, 21–24). Notably, most of these studies have been conducted in Australia, the United States, and European countries, with limited studies conducted among Chinese populations.

To inform the development of effective local tobacco control strategies, it is essential to understand Chinese smokers’ perceptions of plain packaging on their smoking intentions. With funding support from the Tobacco and Alcohol Control Office, Department of Health, Hong Kong Special Administrative Region, we conducted a cross-sectional survey to evaluate current adult smokers’ perceptions of plain versus branded cigarette packs, which both feature 85% PHWs. Specifically, we assessed the perceived attractiveness, harmfulness, and noticeability of PHWs associated with plain versus branded cigarette packs, compared differences in packaging perceptions by smokers’ varying characteristics, evaluated the perceived influences of plain packaging on smoking intentions and behaviours including views on plain pack’s role in deterring youth smoking initiation, and assessed public support for the implementation of plain packaging legislation. It is important to note that these hypotheses concern adults’ perceptions and beliefs about the policy’s impact on youth, not the direct responses of youth, which remains a distinct and valuable area for future research.

## 2 Materials and methods

### 2.1 Study design and participants

This cross-sectional study was conducted from August to November 2023 in Hong Kong. Quantitative and qualitative data were concurrently collected using a structured survey questionnaire developed for this study. Informed consent was obtained from each participant. Ethics approval was obtained from the Joint Chinese University of Hong Kong - New Territories East Cluster Clinical Research Ethics Committee (IRB reference number: 2023.229) and the Survey and Behavioural Research Ethics Committee of The Chinese University of Hong Kong (Reference No. SBRE - 22 - 0707). This study was reported in accordance with the Strengthening the Reporting of Observational Studies in Epidemiology (STROBE) guideline.

Two sets of cigarette packs were prepared for comparison: (1) four existing branded packs with PHWs covering 85% of the pack and (2) four dummy plain packs with PHWs covering 85% of the packs. These four brands were selected based on their popularity and eye-catching designs to ensure their generalizability, while the drab brown (i.e., Pantone 448-C) plain packs used the same colour adopted by most countries that have implemented plain packaging regulations (25). The brands and variant names used for the plain packs were standardised based on Australian cigarette pack design requirements (26). Among the 12 PHWs currently in use in Hong Kong, the warning ‘Smoking takes away my voice’ was chosen for selected cigarette packs. Previous studies have shown that PHWs depicting respiratory disease were the most effective and applicable to both male and female smokers (15). The 12 PHWs were listed on a laminated plastic leaflet, which was shown to the participants to communicate the health risks of smoking.

Current smokers aged 18 years and older who could speak Cantonese or Putonghua were recruited from outdoor smoking hotspots (e.g., mass transit railway station exits, outdoor bus stops, restaurant and shopping plaza entrances) on Hong Kong Island, Kowloon, and the New Territories (27). Time-interval sampling was employed to allow sufficient time (predetermined as 5 min) for interviewers to observe the potential participants’ tobacco use behaviours. Each participant was shown a branded pack and dummy of a plain pack of a randomly selected brand. The research staff then conducted face-to-face interviews with the participants using a structured questionnaire for data collection. After each interview, the interviewers debriefed each participant on the harms of smoking by distributing a printed leaflet presenting the methods for quitting and quitline information.

### 2.2 Measures

The participants were asked to indicate their perceptions of branded and plain cigarette packs with 85% PHWs according to their attractiveness, noticeability of PHWs, cognitive reactions, behavioural responses and support for plain packaging. Demographic characteristics were also collected.

#### 2.2.1 Pack attractiveness

The participants were asked which pack they considered more attractive for purchasing and to rate its attractiveness using a 11-point Likert scale (0 = not attractive at all, 10 = very attractive). They were then invited to provide qualitative feedback to explain their ratings.

#### 2.2.2 Noticeability of pictorial health warnings

The participants identified which pack had more noticeable PHWs and provided the reasons for their choices.

#### 2.2.3 Cognitive reactions

The participants were asked (1) whether the plain packaging appeared more disgusting than the branded packaging, along with an explanation of their reasoning; (2) whether the plain pack made them think more about the health risks of smoking and to explain their reasoning; (3) to rate their agreement with the statement, ‘plain packs convey more severe smoking harms’, using a 5-point Likert scale (1 = strongly disagree to 5 = strongly agree), and (4) whether the plain packs were perceived as less enjoyable and to explain their reasoning.

#### 2.2.4 Behavioural responses

The participants were asked (1) whether the plain packaging would reduce their own intentions to smoke and to explain their reasons and (2) to rate their agreement with the statement, ‘the plain pack will deter new or teenage smokers’, using a 5-point Likert scale (1 = strongly disagree to 5 = strongly agree).

#### 2.2.5 Support for plain packaging

The participants rated their agreement with the statement ‘the government should adopt plain packaging for public health benefits’ using a 5-point Likert scale (strongly disagree to strongly agree).

#### 2.2.6 Other data

The participants’ demographic information and smoking status were also collected. A simplified version of the Fagerstrom test for nicotine dependence (FTND) was used to assess the participants’ nicotine dependence, in which participants with FTND scores of 0–2 were defined as having low nicotine dependence and 3–6 were defined as having moderate-to-high nicotine dependence.

### 2.3 Data analysis

The quantitative data were weighted by sex and age to represent the corresponding population of current smokers in reference to Thematic Household Survey Report (28). Statistical analyses were performed using SPSS software (v.26; IBM SPSS, Armonk, NY, United States). Continuous variables were presented as mean ± standard deviation (SD) and categorical variables were expressed as frequencies with percentages. Using Chi-square test, the participants’ perceptions of cigarette packaging were compared according to their sex, age, education level, nicotine dependence and cigarette brand preference. The qualitative data were extracted and reviewed by two researchers independently to generate codes, and thematic analysis was used to obtain the relevant themes from the participants’ responses. Disagreement between researchers’ code and themes were further discussed to reach agreement.

## 3 Results

A total of 1,256 eligible smokers participated in this study, yielding a response rate of 21.4%. After weighting, the sample was predominately male (71.9%; Table 1). The age distribution was 26.3% (18–39 years), 48.4% (40–59 years), and 25.4% (≥60 years). Over half of the participants (56.7%) had a secondary education, and 31.0% had a tertiary education. Nearly half (46.8%) smoked 11–20 cigarettes per day, while 39.4% smoked 1–10 and 12.3% smoked ≥21 cigarettes per day. Nicotine dependence was low in 50.8% and moderate to high in 49.2% of the participants. Only 28.0% had made quit attempts in the past 12 months, and 26.0% indicated intention to quit in the next 6 months.

**Table 1:** Socio-demographic and smoking characteristics of participants.

Variables	*n* (%)^b^	Weighted n(%)^b^
Sex		
Male	900 (71.8)	910 (71.9)
Female	353 (28.2)	356 (28.1)
Age		
18–39	627 (50.0)	333 (26.3)
40–59	519 (41.4)	613 (48.4)
60 and above	107 (8.5)	321 (25.4)
Education level		
Primary or below	82 (6.5)	155 (12.3)
Secondary	658 (52.6)	717 (56.7)
Tertiary	512(40.9)	392 (31.0)
Cigarettes per day		
≤1	27 (2.2)	18 (1.4)
1–10	530 (42.5)	498 (39.4)
11–20	558 (44.7)	591 (46.8)
≥21	132 (10.6)	155 (12.3)
Nicotine dependence (HSI score)^a^		
Low (0–2)	696 (55.8)	642 (50.8)
Moderate to high (3–6)	551 (44.2)	620 (49.2)
Quit attempt within 12 months		
Yes	359 (28.7)	355 (28.0)
No	893 (71.3)	911 (72.0)
Readiness to quit in 6 months		
Yes	340 (27.2)	328 (26.0)
No	911 (72.8)	936 (74.0)

^a^HIS denotes Heaviness of Smoking Index. ^b^Sum of percentage may not add up to 100% due to rounding.

### 3.1 Quantitative findings

Table 2 presents the quantitative survey findings. The majority of participants (79.8%) agreed that, compared with branded packs, plain packs were less attractive for purchasing. In addition, plain packs were less attractive (weighted mean = 3.5, SD = 2.5) than branded packs (weighted mean = 5.9, SD = 2.5). A higher proportion of participants considered the PHWs on plain packs to be more noticeable than the PHWs on branded packs (57.5% vs. 42.5%, respectively). The mean (SD) values represent participants’ ratings of pack attractiveness on an 11-point Likert scale (0 = “not attractive at all” to 10 = “very attractive”). Higher scores therefore indicate greater perceived attractiveness.

**Table 2:** Perceptions toward pack characteristics and graphic health warnings of the plain and branded packaging (*n* = 1,256).

Perceptions	*n* (%)	Weighted *n* (%)
Attractiveness of pack packaging, mean (SD)		
Branded packs	5.8 (2.5)	5.9 (2.5)
Plain packs	3.6 (2.5)	3.5 (2.5)
Perceived branded/plain packs as more attractive		
Branded packs	960 (76.6)	1,011 (79.8)
Plain packs	293 (23.4)	256 (20.2)
Perceived PHWs as more noticeable		
Branded packs	536 (42.8)	538 (42.5)
Plain packs	717 (57.2)	729 (57.5)
Perceived plain packs as more disgusting		
Yes	533 (43.0)	562 (45.1)
No	707 (57.0)	685 (54.9)
Associated plain packs with more health risks of smoking		
Yes	326 (26.1)	362 (28.7)
No	923 (73.9)	900 (71.3)
Perceived plain packs to convey more severe smoking harms		
Very agree/agree	324 (25.9)	355 (28.0)
No comments	207 (16.5)	222 (17.5)
Very disagree/disagree	722 (57.6)	689 (54.4)
Perceived plain packs as less enjoyable		
Yes	165 (13.2)	189 (15.0)
No	1,085 (86.8)	1,075 (85.0)
Perceived plain packs to reduce smoking intentions		
Yes	199 (15.9)	218 (17.3)
No	1,050 (84.1)	1,042 (82.7)
Perceived plain packs to deter new or teenage smokers		
Agree/strongly agree	402 (32.1)	420 (33.1)
No comments	166 (13.2)	186 (14.7)
Disagree/strongly disagree	685 (54.7)	661 (52.2)
Support for plain packs		
Support/strongly support	428 (34.2)	452 (35.7)
No comments	417 (33.3)	437 (34.5)
Against/strongly against	408 (32.6)	378 (29.9)

^a^PHWs: pictorial health warnings. ^b^The number of cases and valid % vary due to missing cases. ^c^The mean (SD) values represent participants’ ratings of pack attractiveness on an 11-point Likert scale (0 = “not attractive at all” to 10 = “very attractive”).

Nearly half of the participants (45.1%) considered plain packs to be more disgusting than branded packs. In terms of harm perceptions, 28.7% associated plain packs more strongly with smoking harms, 28.0% agreed plain packs conveyed more severe smoking harms and 15% believed that smoking cigarettes from plain packs would be less enjoyable. The perceived behavioural impact of plain packaging was modest: 33.1% believed it could deter new or teenage smokers and 17.3% reported reduced intention to smoke. Considering their support for plain packaging, 35.7% of participants supported the policy, 29.9% opposed it and 34.5% were neutral.

The comparisons of perceptions by participant characteristics showed that lower attractiveness toward plain packs was significantly associated with older age (Supplementary Table S1). Likewise, perceiving plain packs as more disgusting than branded packs was significantly associated with older age and a lower level of education. Greater salience of PHWs on plain packs was significantly associated with being female gender (Supplementary Table S2). Greater perceived harmfulness toward plain packs was associated with older age and a lower level of education. Similarly, greater perceived seriousness of smoking harms toward plain packs was associated with older age.

Beyond statistical differences, several percentage differences demonstrate practical public health significance. The large majority finding plain packs less attractive (~80%) and the majority perceiving PHWs as more noticeable (~58%) reflect meaningful perceptual shifts with established links to reduced tobacco appeal. Although only a smaller proportion reported reduced smoking intention (17.3%), this level is consistent with behavioural outcomes in similar short-exposure studies and still represents a meaningful subgroup likely to benefit from sustained exposure.

### 3.2 Qualitative findings

Thematic analysis revealed five key themes of smokers’ perceptions toward plain packaging (Table 3): preferred to purchase branded packs; negative perceptions and limited positive perceptions toward plain packs; enhanced harm perceptions from plain packs; and perceived smoking experiences and intentions to smoke related to plain packaging.

**Table 3:** Themes from semi-structured interviews on reasons of different perceptions on plain packs.

Themes	Subthemes
1. Preferred to purchase branded packs	1.1 Higher perceived attractiveness of branded packs (e.g., colourful design, brand logos) 1.2 Stronger brand identification and taste association
2. Negative perceptions toward plain packs	2.1 Lower attractiveness (e.g., “dark color,” “lack of design”) 2.2 Disgust triggered by dark color (e.g., associations with death/disease) 2.3 Perceived poorer quality (e.g., “resembles counterfeit cigarettes”)
3. Limited positive perceptions toward plain packs	3.1 Minimalist design appreciated as “high class” 3.2 Dark color evoked perceptions of stronger tobacco strength
4. Enhanced harm perceptions from plain packs	4.1 Higher perceived noticeability of pictorial health warnings (PHWs) 4.2 Heightened smokers’ awareness of smoking-related harms. 4.3 Conveyed a more serious risk messaging of smoking
5. Perceived smoking experiences and intention to smoke related to plain packs	5.1 Reduced smoking enjoyment (e.g., “mood affected by packaging”) 5.2 Decreased purchase/intention to smoke (e.g., due to inconvenience or negative perceptions)

#### 3.2.1 Preferred to purchase branded packs

The participants demonstrated a preference for purchasing branded packs, with visual design elements emerging as the primary differentiator. The appeal of colourful branding was emphasised, where the aesthetic superiority of traditional packaging was described using terms like ‘eye-catching colours’ and ‘vibrant designs’. Brand identification emerged as particularly salient, with participants noting the importance of distinctive logos and clear typography for both product recognition and taste expectations.

#### 3.2.2 Negative perceptions toward plain packs

The participants’ negative attitudes toward plain packs were clustered around three key aspects: the standardised olive-brown colour, minimalist design, and perceived quality. Many respondents associated the drab hue with negative health outcomes and frequently drew parallels to pulmonary disease and mortality. The absence of branding elements led several participants to characterise plain packs as ‘generic’ or ‘counterfeit-like’, with some expressing concerns about reduced product quality despite identical manufacturing standards for the enclosed cigarettes. Even though the branded packs and dummy plain packs were made of the same materials, a few participants considered the plain packs to have poorer quality than branded packs, that is, they considered the plain packs could be ripped easily or the enclosed cigarettes may be fake. Two participants even thought that the taste of cigarettes in plain packs may change, making smoking less enjoyable. These negative perceptions of plain packaging support policy makers to implement this policy to adopt this strategy as a means of reducing smokers’ intention to purchase cigarettes.

#### 3.2.3 Limited positive perceptions toward plain packs

A minority appreciated the minimalist approach, with some describing plain packs as ‘sleek’ or ‘sophisticated’. A subset of smokers, particularly those preferring stronger tobacco, interpreted the dark coloration as indicative of product potency.

#### 3.2.4 Enhanced harm perceptions from plain packs

The analysis revealed plain packaging’s significant impact on the noticeability of PHWs and harm perceptions. Misleading imagery and distractions were eradicated when brand logos were removed from plain packs, which left consumers’ focus on the PHWs. As the PHWs depicted smoking harms, the participants reported that they thought about the harms of smoking more frequently compared with branded packs. Participants consistently noted the enhanced noticeability of PHWs, attributing this to both the increased colour contrast and the elimination of competing visual elements. Many described an amplification effect in which the PHWs appeared more graphic or larger than those on branded packs, which may explain why participants associated plain packs with the harms of smoking more.

#### 3.2.5 Perceived smoking experiences & intention to smoke related to plain packs

Many participants described their reduced motivation to smoke cigarettes, which was attributed to either the unpleasant aesthetics or heightened salience of health risks when smoking cigarettes from plain packs. Practical concerns emerged regarding product differentiation, with some anticipating difficulties in identifying their preferred tobacco varieties when using plain packaging.

Beyond describing perceptions, the themes reflect recognised behavioural pathways. Negative affective responses (e.g., disgust, reduced enjoyment) correspond to affect-driven deterrence mechanisms, while the heightened noticeability of PHWs illustrates increased cue salience, drawing attention toward risk information rather than branding. These cognitive–affective processes are consistent with theoretical models of behaviour change and provide explanatory depth for the observed perceptual and motivational shifts.

## 4 Discussion

This study explored Hong Kong adult smokers’ perceptions of plain packaging, its perceived effectiveness in reducing cigarette consumption, and their support for the policy. As observed in previous research (15, 18, 21, 29), most participants found plain packs with 85% PHWs less attractive than existing branded packs with 85% PHWs. The negative perceptions toward plain packs may be explained by several factors. First, the participants often associated the standardised olive-brown colour with the harmful effects of smoking, including pulmonary diseases and death. Second, the participants believed cigarettes in plain packs had lower quality and poorer taste compared with cigarettes in branded packs and therefore were unlikely to buy plain packs. In contrast, branded packs were perceived as more attractive due to their colourful ‘eye-catching’ designs and logos, which reinforced brand identification and taste expectations. Furthermore, some participants believed that plain packs contained cigarettes with stronger tobacco. Previous study also found that plain packaging was associated with an increased perception of tar content, which might convey serious health risks of smoking more (15). However, whether such negative perceptions deter smokers remains unknown. For example, a focus group study found that heavier smokers believed that plain packaging would not have any impact on their smoking behaviours (30). These findings suggest that plain packaging might not deter heavier smokers and those who prefer stronger tobacco. Our study also found that older participants (i.e., aged ≥60 years) tended to have more negative perceptions toward plain packaging. One plausible hypothesis for this observation could be that socioeconomically disadvantaged smokers, who often experience poorer health outcomes, perceive plain packaging as less attractive and report lower purchase intentions (19). Alternatively, other factors not measured in this study, such as stronger brand attachment formed over a longer smoking history or generational (cohort) effects, could also contribute to this generational difference in perception. Throughout, we interpret findings as perceived or self-reported impacts following brief exposure, rather than demonstrated changes in smoking behaviour.

In the present study, more than half of the participants found PHWs to be more noticeable on the plain packs compared to branded packs, despite the PHWs being identical in size and design, suggesting that the standardised pack design may draw attention toward PHWs and away from branding elements. This finding is supported by eye-tracking studies, which have shown that both adults and adolescents fixate more on PHWs when viewing plain versus branded packs (20). The increased salience of PHWs more effectively communicated the harmful effects of tobacco use, thereby negatively influencing smokers’ perception of the enclosed cigarettes. Our finding reinforces the literature showing that combining plain packaging with PHWs may produce a synergistic effect, leading to further negative perceptions toward plain packs and reduced pack attractiveness (15).

Some participants reported that plain packs would reduce their intention to smoke. While this figure may appear modest, it is likely influenced by their brief exposure to plain packaging during the study. Evidence indicates that the impact of plain packaging on quit intentions is more pronounced with prolonged exposure – typically 1 week or longer – whereas studies involving shorter exposure durations often report limited or no significant effects (23). Therefore, the effectiveness of plain packaging may be underestimated in short-term assessments. Nonetheless, our findings on initial perceptions provide a plausible precursor to the long-term behavioural outcomes documented after implementation in other jurisdictions. For example, research in Australia attributed approximately one-quarter of the decline in smoking prevalence over 3 years to plain packaging (14), while in France, 22.5% of ex-smokers reported it as a quit motivator within 1 year (22). While distinct in design (perceptual vs. behavioural), our study offers the kind of early, stakeholder-specific evidence that can inform the pre-implementation policy debate, much as post-implementation data guide subsequent evaluation.

Approximately one-third of respondents believed that plain packaging would deter new and teenage smokers. This is particularly important, as younger individuals are more likely to be influenced by the visual appeal of colourful packaging (15). By eliminating these design elements, plain packaging reduces the ability of tobacco companies to attract new users through misleading branding, which downplays the harms of smoking. Previous studies have similarly reported the effectiveness of plain packaging in discouraging smoking initiation among non-smokers, particularly youth (21). Collectively, these findings support the adoption of plain packaging to encourage smoking cessation and prevent smoking initiation.

Over half of the participants reported smoking more than 11 cigarettes per day, and nearly half exhibited moderate-to-high levels of nicotine dependence. Despite this significant dependence, many smokers perceived plain packaging as reducing the attractiveness of cigarette packs and enhancing the visibility of PHWs. Notably, more than two-thirds of the participants either supported or were neutral toward the implementation of plain packaging legislation in Hong Kong. Given that public support for plain packaging is typically higher in the general population than among current smokers, it is reasonable to expect even broader acceptance of this measure beyond the study sample. The findings suggested that the social and policy environment in Hong Kong was conducive to considering plain packaging legislation. Collectively, the results of this large-scale survey provided timely empirical evidence that can inform policy deliberations regarding the adoption of plain packaging. The presentation of the study findings by the Department of Health formed part of ongoing public health communication activities. The subsequent enactment of the 2025 tobacco control legislation reflects the government’s broader policy agenda rather than a direct outcome of this study. Our findings should be understood as evidence that may inform policy discussions, not as determinants of legislative change.

Policy implications.

In alignment with the WHO’s 2025 World No Tobacco Day, the Department of Health of Hong Kong SAR launched a public campaign and held a press briefing on 23 May 2025 to present the study findings. Following the collective efforts of stakeholders across various community sectors, the Tobacco Control Legislation (Amendment) Bill 2025 was successfully enacted on 19 September 2025, and the implementation of plain packaging is scheduled to take effect in the second quarter of 2027 (31).

This study has several limitations. First, the sampling strategy of recruiting participants from outdoor smoking hotspots may introduce selection bias. Smokers who frequently appeared at these locations might differ systematically (e.g., in smoking intensity, social habits, or daily routines) from those who do not, potentially limiting the generalizability of our findings to the broader population of adult smokers in Hong Kong. The modest response rate (21.4%) is consistent with challenges in recruiting for health-related surveys in public spaces but further underscores that our sample may not be fully representative. Consequently, our estimates of support for or perceptions of plain packaging should be interpreted with this potential bias in mind. Future studies could employ random digit dialing, household surveys, or targeted online panels to achieve a more representative sample. Second, the experimental exposure to plain packaging was brief, participants provided immediate perceptual responses in a structured interview setting. While this method is standard and valid for assessing initial cognitive reactions (such as perceived attractiveness and the salience of health warnings), it cannot capture how perceptions might evolve with prolonged, real-world exposure over weeks or months. Nor does it measure actual changes in smoking behaviors. Therefore, our findings should be framed as evidence of plain packaging’s potential to alter initial impressions, which is a critical first step in its hypothesized causal pathway. The translation of these immediate perceptions into sustained attitudinal or behavioral change requires confirmation through longitudinal studies or natural experiments in jurisdictions that have implemented plain packaging. Third, the participants’ responses may have been influenced by social desirability bias. Given that plain packaging is a government-proposed public health measure, some participants might have felt inclined to report more favourable attitudes toward it, perceiving this as the socially or politically ‘correct’ stance. This could lead to an overestimation of true support levels in the population. The street-intercept interview method, while practical, involves face-to-face interaction which may amplify this bias compared to fully anonymous surveys. Consequently, while the reported perceptions of reduced pack attractiveness and increased warning salience are less susceptible to this bias, the quantitative estimates of support for the policy (e.g., the 35.7% in favour) should be interpreted as potentially representing an upper bound of public acceptance. Future research could employ methods such as anonymous online surveys or incorporate indirect questioning techniques to help mitigate this bias and obtain more robust estimates of policy support. The fourth limitation was the use of bivariate statistics (e.g., chi-square tests) to describe associations and differences in perceptions. While this approach is appropriate and clearly demonstrates the unadjusted relationships between pack type and smoker perceptions, it does not account for potential confounding variables. Factors such as smoking intensity (e.g., cigarettes per day), nicotine dependence, education level, or prior quit attempts could influence both how a smoker perceives plain packaging and their reported behavioral intentions. Future research should employ multivariable regression models by controlling for such potential confounders, thereby providing more robust estimates of its impact. Finally, these findings reflect the perceptions of smokers within the specific context of Hong Kong, a high-density urban setting with an established 85% PHW policy. This cultural and regulatory specificity should be considered when generalizing the results to other regions. Our study thus offers a valuable reference point for understanding smoker responses in markets with comparable public health regimes. These findings offer valuable preliminary evidence that supports the implementation of plain packaging in Hong Kong. Future research is needed to evaluate the real-world, long-term effects of plain packaging on smoking behaviour and smoking cessation outcomes. Lastly, we acknowledge that using single-item measures may not fully capture the multidimensional nature of constructs such as disgust or enjoyment and may limit reliability compared with multi-item scales. Future research could incorporate validated multi-item affective or perceptual scales to improve measurement precision and enhance psychometric robustness.

## 5 Conclusion

This study found that adult smokers in Hong Kong perceive plain packaging as less attractive, more effective in highlighting health warnings, and potentially useful in deterring smoking initiation, particularly among youth. These perceptions provide critical, stakeholder-informed evidence for policymakers considering plain packaging as a complement to existing measures such as taxation, cessation services, and public education. Hong Kong’s experience, documented here through smoker perspectives, may offer a valuable reference for other jurisdictions evaluating this policy within a comprehensive tobacco control strategy.

## Data Availability

The raw data supporting the conclusions of this article will be made available by the authors, without undue reservation.
