# Supplementary material for: Strengthening tobacco control policy: using plain packaging to reduce product appeal and enhance public awareness
Source: Front Public Health. 2026 Mar 9;14:1781256. doi: 10.3389/fpubh.2026.1781256 (PMC13006678; doi:10.3389/fpubh.2026.1781256)
Supplement: Supplementary file 1 [file Table_1.DOCX]

Table S1. Attractiveness towards branded and plain packs by socio-demographics and smoking status in adult smokers (N=1256)

|  | Perceived branded packs as more attractive | | Perceived plain packs as more attractive | |  | Perceived plain packs as more disgusting | | Did not perceive plain packs as more disgusting | |  |
| --- | --- | --- | --- | --- | --- | --- | --- | --- | --- | --- |
|  | N ^a^ | Weighted % | N ^a^ | Weighted % | P value ^b^ | N ^a^ | Weighted % | N ^a^ | Weighted % | P value ^b^ |
| Sex |  |  |  |  |  |  |  |  |  |  |
| Male | 685 | 80.4 | 167 | 19.6 | 0.601 | 392 | 43.9 | 500 | 56.1 | 0.171 |
| Female | 268 | 79.1 | 71 | 20.9 |  | 167 | 48.3 | 179 | 51.7 |  |
| Age |  |  |  |  |  |  |  |  |  |  |
| 18-39 | 232 | 73.4 | 84 | 26.6 | <0.001 | 142 | 43.0 | 188 | 57.0 | 0.046 |
| 40-59 | 464 | 79.5 | 120 | 20.5 |  | 260 | 43.0 | 344 | 57.0 |  |
| 60 and above | 260 | 87.2 | 38 | 12.8 |  | 160 | 51.1 | 153 | 48.9 |  |
| Education level |  |  |  |  |  |  |  |  |  |  |
| Primary or below | 107 | 76.4 | 33 | 23.6 | 0.083 | 70 | 46.7 | 80 | 53.3 | 0.013 |
| Secondary | 558 | 81.9 | 123 | 18.1 |  | 341 | 48.2 | 367 | 51.8 |  |
| Tertiary | 288 | 76.8 | 87 | 23.2 |  | 150 | 39.0 | 235 | 61.0 |  |
| Nicotine dependence  (HSI score) |  |  |  |  |  |  |  |  |  |  |
| Low (0–2) | 477 | 78.5 | 131 | 21.5 | 0.199 | 288 | 45.1 | 351 | 54.9 | 0.964 |
| Moderate to high (3–6) | 478 | 81.4 | 109 | 18.6 |  | 273 | 45.2 | 331 | 54.8 |  |
| Brand of cigarette interviewed |  |  |  |  |  |  |  |  |  |  |
| Marlboro Red | 419 | 75.6 | 135 | 24.4 | 0.009 | 251 | 44.3 | 315 | 55.7 | 0.007 |
| Mevius Max Yellow | 261 | 84.7 | 47 | 15.3 |  | 133 | 42.5 | 180 | 57.5 |  |
| Chesterfield Menthol | 157 | 82.6 | 33 | 17.4 |  | 118 | 55.1 | 96 | 44.9 |  |
| Pall Mall Blue | 119 | 81.0 | 28 | 19.0 |  | 60 | 38.7 | 95 | 61.3 |  |

Sum of percentage may not add up to 100% due to rounding.

^a^ The total number of subjects differed due to missing data.

^b^ calculated by Chi-square test.
