# Supplementary material for: Strengthening tobacco control policy: using plain packaging to reduce product appeal and enhance public awareness
Source: Front Public Health. 2026 Mar 9;14:1781256. doi: 10.3389/fpubh.2026.1781256 (PMC13006678; doi:10.3389/fpubh.2026.1781256)
Supplement: Supplementary file 2 [file Table_2.DOCX]

Table S2 Perceived harmfulness towards branded and plain packs by socio-demographics and smoking status in adult smokers (N=1256)

|  | Perceived health warnings on branded packs as more salient | | Perceived health warnings on plain packs were more salient | |  | Associated plain packs with the harm of smoking more | | Did not associate plain packs with the harm of smoking more | |  | Associated plain packs with more serious health risk | | Did not associate plain packs with more serious health risk | |  |
| --- | --- | --- | --- | --- | --- | --- | --- | --- | --- | --- | --- | --- | --- | --- | --- |
|  | N ^a^ | Weighted % | N ^a^ | Weighted % | P value ^b^ | N ^a^ | Weighted % | N ^a^ | Weighted % | P value ^b^ | N ^a^ | Weighted % | N ^a^ | Weighted % | P value ^b^ |
| Sex |  |  |  |  |  |  |  |  |  |  |  |  |  |  |  |
| Male | 347 | 44.7 | 430 | 55.3 | 0.027 | 259 | 28.9 | 637 | 71.1 | 0.678 | 257 | 28.5 | 644 | 71.5 | 0.630 |
| Female | 116 | 37.3 | 195 | 62.7 |  | 99 | 27.7 | 258 | 72.3 |  | 97 | 27.2 | 260 | 72.8 |  |
| Age |  |  |  |  |  |  |  |  |  |  |  |  |  |  |  |
| 18-39 | 135 | 45.3 | 163 | 54.7 | 0.267 | 79 | 23.8 | 253 | 76.2 | <0.001 | 80 | 24.0 | 253 | 76.0 | <0.001 |
| 40-59 | 208 | 39.9 | 313 | 60.1 |  | 158 | 25.8 | 454 | 74.2 |  | 156 | 25.4 | 457 | 74.6 |  |
| 60 and above | 122 | 44.0 | 155 | 56.0 |  | 125 | 39.3 | 193 | 60.7 |  | 119 | 37.1 | 202 | 62.9 |  |
| Education level |  |  |  |  |  |  |  |  |  |  |  |  |  |  |  |
| Primary or below | 55 | 40.7 | 80 | 59.3 | 0.294 | 48 | 31.6 | 104 | 68.4 | <0.001 | 45 | 29.0 | 110 | 71.0 | 0.469 |
| Secondary | 257 | 41.1 | 369 | 58.9 |  | 232 | 32.4 | 484 | 67.6 |  | 209 | 29.1 | 508 | 70.9 |  |
| Tertiary | 153 | 46.1 | 179 | 53.9 |  | 82 | 21.0 | 309 | 79.0 |  | 101 | 25.8 | 291 | 74.2 |  |
| Nicotine dependence  (HSI score) |  |  |  |  |  |  |  |  |  |  |  |  |  |  |  |
| Low (0–2) | 226 | 40.5 | 332 | 59.5 | 0.163 | 190 | 29.6 | 451 | 70.4 | 0.501 | 196 | 30.5 | 446 | 69.5 | 0.060 |
| Moderate to high (3–6) | 239 | 44.7 | 296 | 55.3 |  | 172 | 27.9 | 444 | 72.1 |  | 160 | 25.8 | 461 | 74.2 |  |
| Brand of cigarette interviewed |  |  |  |  |  |  |  |  |  |  |  |  |  |  |  |
| Marlboro Red | 198 | 39.7 | 301 | 60.3 | 0.004 | 136 | 23.4 | 446 | 76.6 | <0.001 | 142 | 24.3 | 442 | 75.7 | 0.003 |
| Mevius Max Yellow | 112 | 38.2 | 181 | 61.8 |  | 102 | 32.5 | 212 | 67.5 |  | 103 | 32.9 | 210 | 67.1 |  |
| Chesterfield Menthol | 85 | 52.8 | 76 | 47.2 |  | 80 | 37.7 | 132 | 62.3 |  | 75 | 34.7 | 141 | 65.3 |  |
| Pall Mall Blue | 71 | 49.3 | 73 | 50.7 |  | 45 | 29.2 | 109 | 70.8 |  | 36 | 23.2 | 119 | 76.8 |  |
| Sum of percentage may not add up to 100% due to rounding. | | | | | | | | | | |  |  |  |  |  |
| ^a^ The total number of subjects differed due to missing data.  ^b^ calculated by Chi-square test. | | | | | | | | | | |  |  |  |  |  |
